# *APOE* ε4 and Intracerebral Hemorrhage in Patients With Brain Arteriovenous Malformation

**DOI:** 10.1001/jamanetworkopen.2023.55368

**Published:** 2024-02-16

**Authors:** Daniela Renedo, Cyprien A. Rivier, Andrew B. Koo, Nanthiya Sujijantarat, Santiago Clocchiatti-Tuozzo, Kane Wu, Victor M. Torres-Lopez, Shufan Huo, Murat Gunel, Adam de Havenon, Kevin N. Sheth, Charles C. Matouk, Guido J. Falcone

**Affiliations:** 1 Department of Neurosurgery, Yale School of Medicine, New Haven, Connecticut; 2 Department of Neurology, Yale School of Medicine, New Haven, Connecticut; 3 Yale Center for Brain and Mind Health, New Haven, Connecticut

## Abstract

**Importance:**

Intracerebral hemorrhage (ICH) is a serious complication of brain arteriovenous malformation (AVM). Apolipoprotein E (*APOE*) ε4 is a well-known genetic risk factor for ICH among persons without AVM, and cerebral amyloid angiopathy is a vasculopathy frequently observed in *APOE* ε4 carriers that may increase the risk of ICH.

**Objective:**

To assess whether *APOE* ε4 is associated with a higher risk of ICH in patients with a known AVM.

**Design, Setting, and Participants:**

This cross-sectional study including 412 participants was conducted in 2 stages (discovery and replication) using individual-level data from the UK Biobank (released March 2012 and last updated October 2023) and the All of Us Research Program (commenced on May 6, 2018, with its latest update provided in October 2023). The occurrence of AVM and ICH was ascertained at the time of enrollment using validated *International Classification of Diseases, Ninth Revision* and *Tenth Revision,* codes. Genotypic data on the *APOE* variants rs429358 and rs7412 were used to ascertain the ε status.

**Main Outcomes and Measures:**

For each study, the association between *APOE* ε4 variants and ICH risk was assessed among patients with a known AVM by using multivariable logistic regression.

**Results:**

The discovery phase included 253 UK Biobank participants with known AVM (mean [SD] age, 56.6 [8.0] years, 119 [47.0%] female), of whom 63 (24.9%) sustained an ICH. In the multivariable analysis of 240 participants of European ancestry, *APOE* ε4 was associated with a higher risk of ICH (odds ratio, 4.58; 95% CI, 2.13-10.34; *P* < .001). The replication phase included 159 participants with known AVM enrolled in All of Us (mean [SD] age, 57.1 [15.9] years; 106 [66.7%] female), of whom 29 (18.2%) sustained an ICH. In multivariable analysis of 101 participants of European ancestry, *APOE* ε4 was associated with higher risk of ICH (odds ratio, 4.52; 95% CI, 1.18-19.38; *P* = .03).

**Conclusions and Relevance:**

The results of this cross-sectional study of patients from the UK Biobank and All of Us suggest that information on *APOE* ε4 status may help identify patients with brain AVM who are at particularly high risk of ICH and that cerebral amyloid angiopathy should be evaluated as a possible mediating mechanism of the observed association.

## Key Points

**Question:**

Are apolipoprotein (*APOE*) ε4 variants associated with the occurrence of intracerebral hemorrhage in patients with brain arteriovenous malformation?

**Findings:**

In a cross-sectional study of 2 different cohorts for a total of 412 patients with known brain arteriovenous malformation, carriers of *APOE* ε4 variants had a 4-fold increase in the risk of sustaining an intracerebral hemorrhage.

**Meaning:**

These findings suggest that information on *APOE* ε4 status may help identify patients with brain arteriovenous malformation who are at particularly high risk of intracerebral hemorrhage.

## Teaser

This cross-sectional study uses UK Biobank and All of Us Research Program data to assess whether APOE ε4 is associated with higher risk of intracerebral hemorrhage among patients with known brain arteriovenous malformation.

## Introduction

Brain arteriovenous malformations (AVMs) are abnormal connections between arteries and veins without an intervening capillary bed.^1^ This direct shunting results in high-pressure blood flow from the arteries through the AVM nidus to draining veins, resulting in localized venous hypertension. Intracerebral hemorrhage (ICH) represents the most common and serious cause of AVM-related morbidity and mortality. Natural history studies of untreated brain AVM have demonstrated an annual risk of ICH of 2% to 4%.^2,3,4^ Patients with a history of a previous bleed have a risk of recurrent bleeding as high as 14% to 20%.^5,6^ There are several recognized risk factors for brain AVM bleeding, such as the presence of associated aneurysms near or within the AVM nidus, deep venous drainage, younger age, and pregnancy.^2,7,8^ However, the mechanisms that lead to ICH in persons with AVM remain incompletely understood. Because whether to aggressively treat these patients remains a controversial topic, the identification of novel risk factors for ICH in this population could help improve existing risk-stratification strategies. Several studies have demonstrated that ε4 variants within the apolipoprotein E (*APOE*) gene lead to higher risk of cerebral amyloid angiopathy and spontaneous ICH among patients who do not have underlying AVM.^9,10^ In this study, we tested the hypothesis that *APOE* ε4 variants are associated with higher risk of ICH among patients with known brain AVM.

## Methods

### Study Design

We conducted a 2-stage (discovery and replication) case-control genetic association study with a cross-sectional design using data from the UK Biobank (released in March 2012 and last updated in October 2023) and from the All of Us Research Program (commenced on May 6, 2018, with its latest update provided in October 2023). In both studies, we compared patients who, at enrollment, had a known AVM and a prior ICH (cases) vs those who had a known AVM but did not have ICH. The UK Biobank is a prospective population-based cohort study that recruited 500 000 community-dwelling individuals aged 40 to 69 years between 2006 and 2010 from across the United Kingdom.^11^ The All of Us Research Program, conducted by the National Institutes of Health, aims to enroll 1 million Americans from demographically, geographically, and medically diverse groups across the United States, with open enrollment to all who choose to participate. The program began national enrollment in 2018 and is expected to last at least 10 years.^12^

Both studies have appropriate institutional review board oversight: the All of Us Research Program receives oversight from the program’s institutional review board; the UK Biobank has approval from the North West Multi-Centre Research Ethics Committee as a Research Tissue Bank (RTB) approval, meaning that researchers do not require separate ethical clearance. All participating persons provided written informed consent. We followed the Strengthening the Reporting of Observational Studies in Epidemiology (STROBE) reporting guideline for cross-sectional studies. See eFigure in Supplement 1 for a detailed study flow diagram.

### Ascertainment of AVM and ICH Occurrence

We ascertained AVM occurrence in study participants enrolled in both studies using the previously validated *International Classification of Diseases, Ninth Revision* and *Tenth Revision* (*ICD-9*, *ICD-10*) codes 747.81 and Q28.2.^13,14,15^ Following prior research, we also used *ICD-9* and *ICD-10* codes (431 and I61.0-I61.9) to ascertain ICH that happened before or after the AVM code appeared in the electronic health records of those participants.^16^

### Genomic Data and *APOE* Status

For the UK Biobank, DNA samples were collected at the time of enrollment and genome-wide data were obtained using the UK Biobank Axiom Array. In the All of Us Research Program, DNA samples are collected via blood and saliva samples obtained at dedicated research centers across the US, and genome-wide data were generated using the Illumina Global Diversity Array.^17^ In both studies, genome-wide data were quality controlled and imputed centrally using standardized pipelines that follow the most up-to-date standards in the field.^17^ Following current standard practices, we used genotypic data on the *APOE* variants rs429358 and rs7412 to ascertain the ε status as *APOE* ε2, ε3, or ε4. Among these, ε3 is the most common and considered the wild type.^18^

### Race and Ethnicity Ascertainment

Race and ethnicity were included in the analyses because *APOE* effect can vary by race. In both studies we used participants’ genetic ancestry. Race and ethnicity categories for the UK Biobank included Caucasian (European ancestry) and for All of Us, the race and ethnicity categories included African or African American, American admixed or Latino; East Asian; European; Middle Eastern; South Asian; and others. For analyses involving all ancestries, all categories were included.

### Statistical Analysis

Discrete variables are presented as counts (percentages) and continuous variables as means (SDs) or medians (IQRs), as appropriate. In the discovery stage, our primary analysis focused on persons of European ancestry and used a previously validated analytical approach that entailed fitting multivariable logistic regression, modeling *APOE* ε4 and ε2 additively (the variable for ε4 and ε2 takes the values of 0, 1, or 2, respectively, and is entered as an ordinal predictor), and adjusting for age, sex, and 4 genetic principal components for ancestry. We used a complete case approach in which analyses include study participants with available data for all the variables of interest. In secondary analyses, we evaluated study participants of all ancestries and incorporated vascular risk factors and comorbidities into our regression models. We also implemented interaction analyses to evaluate whether age and sex modified the observed associations. In the replication stage, we recapitulated the primary analysis conducted for the discovery phase. Secondary analyses with multivariable models were also used for participants of all ancestries and in sensitivity analysis looking at incident and prevalence cases (eTables 2, 3, 4, and 5 in Supplement 1). We used R (R Project for Statistical Computing) for all statistical analyses and PLINK, version 1.9,^19^ for genetic analyses. A 2-sided value of *P* < .05 was considered statistically significant.

## Results

The latest data release for the UK Biobank included 502 442 study participants, and for All of Us, 372 397 study participants. Of these participants, 487 442 for UK Biobank and 98 622 for All of Us had available genomic data and passed quality control filters. Across both studies, there were 412 participants with AVM, including 253 from the UK Biobank and 159 from All of Us (Table 1).

**Table 1.** Characteristics of the Study Population

Characteristic	UK Biobank participants, No (%) (n = 253)	All of Us participants, No (%) (n = 159)
Concomitant brain AVM plus ICH	63 (24.9)	29 (18.2)
Age, mean (SD), y	56.6 (8.0)	57.1 (15.9)
Sex		
Female	119 (47.0)	106 (66.7)
Male	134 (53.0)	53 (33.3)
Ancestry		
European	240 (95.2)	101 (63.5)
Non-European	<20^a^	58 (36.5)
Vascular risk factors		
Hypertension	87 (34.4)	<20^a^
Hyperlipidemia	76 (30.0)	<20^a^
Diabetes	30 (11.9)	<20^a^
Smoking	140 (55.3)	74 (46.4)
*APOE* ε4 (1 or 2 alleles)	71 (28.1)	31 (19.5)
*APOE* ε2 (1 or 2 alleles)	30 (11.9)	24 (15.1)

Abbreviations: AVM, arteriovenous malformation; ICH, intracerebral hemorrhage.

^a^ Data are redacted for privacy reasons.

### Discovery Stage

In the discovery phase conducted using the UK Biobank, there were 253 participants with brain AVM (mean [SD] age, 56.6 [8.0] years; 119 [47.0%] female, and 134 [53.0%] male), of whom 63 (24.9%) had concomitant ICH (Table 1). Our primary analyses focused on 240 participants of European ancestry, including 59 participants (24.5%) who had an ICH. In this study population, each additional *APOE* ε4 allele was associated with a 4-fold increase in the risk of ICH (odds ratio [OR], 4.58; 95% CI, 2.13-10.34; *P* < .001) (Table 2). These results remained significant in secondary analyses that included participants of all race and ethnicity groups and evaluated the addition of vascular risk factors and comorbidities to regression models (eTable 1 in Supplement 1). Age and sex did not significantly modify the observed association. The *APOE* ε2 variants were not associated with a higher risk of intracranial bleeding (OR, 1.57; 95% CI, 0.50-4.17; *P* = .42) (Table 2).

**Table 2.** Association Analyses Between *APOE* ε Status and Risk of Intracerebral Hemorrhage Among Patients of European Ancestry With Brain Arteriovenous Malformation

*APOE* status	Discovery phase, UK Biobank				Replication phase, All of Us			
	Univariate logistic regression		Multivariable logistic regression		Univariate logistic regression		Multivariable logistic regression	
	OR (95% CI)	*P* value	OR (95% CI)	*P* value	OR (95% CI)	*P* value	OR (95% CI)	*P* value
*APOE* ε4	3.94 (1.95-8.17)	<.001	4.58 (2.13-10.34)	<.001	3.75 (1.25-11.46)	.01	4.52 (1.18-19.38)	.03
*APOE* ε2	0.82 (0.28-1.95)	.51	1.57 (0.50-4.17)	.42	0.25 (0.01-1.29)	.20	0.32 (0.01-1.74)	.36

Abbreviation: OR, odds ratio.

### Replication Stage

In the replication stage conducted using All of Us, there were 159 participants with brain AVM (mean [SD] age, 57.1 [15.9] years; 106 [66.7%] female, and 53 [33.3%] male), of whom 29 (18.2%) had an ICH (Table 1). Of these 159 participants, 101 (60.5%) were of European ancestry, and 58 (36.5%) were of other ancestries. The primary replication analysis focused on participants of European ancestry and confirmed that each additional ε4 allele was associated with a 4-fold increase in the risk of ICH (OR, 4.52; 95% CI, 1.18-19.38; *P* = .03) (Table 2).

## Discussion

This cross-sectional study leveraged 2 landmark population studies undertaken in 2 different countries to conduct a 2-stage genetic association study aimed at evaluating the role of *APOE* ε variants in persons with known brain AVM. We found that persons carrying *APOE* ε4 variants had a 4-fold higher risk of sustaining an ICH. These results remained significant across secondary analyses designed to tackle possible sources of bias. In addition, the effect estimates were consistent across the 2 evaluated studies.

The *APOE* ε4 variants are well-established genetic risk factors for ICH related to cerebral small vessel disease, particularly in the context of cerebral amyloid angiopathy. Given this background, our focus was on brain AVM to assess the role of these variants in the clinical trajectory of such patients, a currently understudied area. While acknowledging the broader implications of *APOE* variants, we delimited our scope to cerebral events, providing a targeted exploration within this specific vascular pathology. By showing that *APOE* ε4 was associated with a significant increase in ICH risk in this specific population of patients, our findings support further research aimed at evaluating the role of cerebral amyloid angiopathy in brain AVM carriers. In addition, our results support the addition of ε4 to the list of known risk factors for brain hemorrhage in these patients, which includes a history of prior bleeding and specific anatomical characteristics of the AVM (small size, deep venous drainage, presence of intranidal aneurysm, and higher feeding artery pressure).^20^ The stratification of ICH risk is of the utmost importance in these patients, as the benefit of invasive neurosurgical interventions (including endovascular treatments) hinge on the a priori risk of sustaining a brain hemorrhage.

A possible biological explanation for the observed finding is the established association between the *APOE* ε4 allele and cerebral amyloid angiopathy.^21^ The deposition of amyloid makes brain vessels frail and vulnerable to hemorrhagic events. An AVM, with its abnormal blood flow, imposes added stress on these vessels. When these genetically fragile vessels, influenced by the *APOE* ε4 allele, encounter the hemodynamic changes typical of AVMs, their risk of rupture and subsequent ICH is significantly increased.

The potential use of *APOE* data to risk stratify persons with AVM is further supported by well-designed studies aimed at evaluating the feasibility and consequences of returning genetic information to patients.^22,23,24^ Those studies found that disclosing the *APOE* ε4 status, which is also a well-established risk factor for Alzheimer disease, led to improvements in beneficial health habits without significant short-term psychological adverse effects.^22^ In addition, direct-to-consumer companies have made this information available to millions of Americans in recent years.

An important next step would be to confirm our findings in a dedicated prospective study. Such study would prioritize the inclusion of patients who have been recently diagnosed with brain AVM. Essential to this research would be the availability of genome-wide data that would allow appropriate consideration of ancestry in addition to *APOE* status. Another important component of such study would be the inclusion of neuroimaging assessments, if possible, evaluating both the brain parenchyma and vasculature. The latter would allow for the evaluation of specific radiologic markers indicative of an increased risk of bleeding. Prospectively confirming the role of the *APOE* variants and the possible synergy of high-risk neuroimaging features could have important clinical implications via identification of patients with AVM at high risk of ICH.

### Strengths and Limitations

An important strength of this work is the use of 2 different population studies that yielded surprisingly consistent estimates for the ε4-related ICH risk in this patient population. One important limitation is that the ascertainment of both AVM and ICH occurrences was based on electronic health record data and *ICD* codes, which are more prone to errors than data from a controlled study setting. Another limitation is the restricted power to appropriately evaluate this question in non–European ancestry populations, which limits the generalizability of these results. To our knowledge, we have reported a greater occurrence of brain AVM than has been documented in the literature, suggesting a possible bias in the way the previous and the present studies were conducted.^25^ This discrepancy may reflect cohort-specific factors, such as differing demographics, health profiles, and the inclusive nature of our data capture, which accounts for both incident and prevalent cases, as well as secondary diagnoses. Such variables, along with the extended temporal scope of our study, likely contributed to the observed increase in prevalence, underscoring the nuanced challenges inherent in accurately determining the prevalence of brain AVM.

## Conclusion

The results of this cross-sectional study involving 253 UK Biobank and 159 All of Us participants with brain AVM suggest that *APOE* ε4 status may be crucial in pinpointing patients with an elevated risk of ICH, particularly those of European ancestry. This study identified cerebral amyloid angiopathy, commonly observed in *APOE* ε4 carriers and known for increasing intracerebral hemorrhage risk, as a potential mediator in this association. This finding underscores the need for further research to validate these results and to explore how *APOE* ε4 information may be integrated into precision medicine approaches for stratifying risk among patients with AVM.

## Notes

Supplement 1. **eTable 1.** Secondary association analyses between *APOE* epsilon 4 and risk of ICH in patients with brain arteriovenous malformations **eTable 2.** Association analyses between *APOE* epsilon status and risk of incident ICH in patients with brain arteriovenous malformations of European ancestry **eTable 3.** Association analyses between *APOE* epsilon status and risk of incident intracerebral hemorrhage in patients with brain AVMs in all ancestries **eTable 4.** Association analyses between *APOE* epsilon status and risk of prevalent ICH in patients with brain arteriovenous malformations of European ancestry **eTable 5.** Association analyses between *APOE* epsilon status and risk of prevalent ICH in patients with brain arteriovenous malformations of all ancestries **eFigure.** Study flow diagram

Supplement 2.
Data Sharing Statement
