# Supplementary material for: APOE ε4 and Intracerebral Hemorrhage in Patients With Brain Arteriovenous Malformation
Source: JAMA Netw Open. 2024 Feb 16;7(2):e2355368. doi: 10.1001/jamanetworkopen.2023.55368 (PMC10873768; doi:10.1001/jamanetworkopen.2023.55368)
Supplement: Supplement 1. — eTable 1. Secondary association analyses between APOE epsilon 4 and risk of ICH in patients with brain arteriovenous malformations eTable 2. Association analyses between APOE epsilon status and risk of incident ICH in patients with brain arteriovenous malformations of European ancestry eTable 3. Association analyses between APOE epsilon status and risk of incident intracerebral hemorrhage in patients with brain AVMs in all ancestries eTable 4. Association analyses between APOE epsilon status and risk of prevalent ICH in patients with brain arteriovenous malformations of European ancestry eTable 5. Association analyses between APOE epsilon status and risk of prevalent ICH in patients with brain arteriovenous malformations of all ancestries eFigure. Study flow diagram [file jamanetwopen-e2355368-s001.pdf]

Supplementary Online Content

Renedo D, Rivier CA, Koo AB, et al. *APOE* $\varepsilon 4$ and intracerebral hemorrhage in patients with brain arteriovenous malformation. *JAMA Netw Open*. 2024;7(2):e2355368. doi:10.1001/jamanetworkopen.2023.55368

eTable 1. Secondary association analyses between *APOE* epsilon 4 and risk of ICH in patients with brain arteriovenous malformations

eTable 2. Association analyses between *APOE* epsilon status and risk of incident ICH in patients with brain arteriovenous malformations of European ancestry

eTable 3. Association analyses between *APOE* epsilon status and risk of incident intracerebral hemorrhage in patients with brain AVMs in all ancestries

eTable 4. Association analyses between *APOE* epsilon status and risk of prevalent ICH in patients with brain arteriovenous malformations of European ancestry

eTable 5. Association analyses between *APOE* epsilon status and risk of prevalent ICH in patients with brain arteriovenous malformations of all ancestries

eFigure. Study flow diagram

This supplementary material has been provided by the authors to give readers additional information about their work.

eTable 1. Secondary association analyses between *APOE* epsilon 4 and risk of ICH in patients with brain arteriovenous malformations

Analysis	UK Biobank			
	Univariate logistic regression		Multivariate logistic regression	
	Odds ratio (95% CI)	p-value	Odds ratio (95% CI)	p-value
All ancestries<br>Adjusting for age, sex and principal components 1-4	1,74 (1.01-2.99)	0.04	1.73 (1.00- 3.03)	0.05
Europeans<br>Adjusting for age, sex, vascular risk factors, comorbidities and principal components 1-4	2.41 (1.29-4.52)	0.01	4.76 (2.17-10.96)	<0.001
All ancestries<br>Adjusting for age, sex, vascular risk factors, comorbidities and principal components 1-4	1,74 (1.01-2.99)	0.04	1.76 (1.01- 3.10)	0.04

*Multivariate analyses adjusted by age, sex, cardiovascular risk factors and and PCA.

*All ancestries: included all ancestries in each study (Categories for UK Biobank includes: Caucasian (European Ancestry) and in All of Us included: African/African American, Amercian/Admixed/Latino, East Asian, European, Middle Eastern, South Asia, and Others.)

© 2024 Renedo D et al. *JAMA Network Open*.

eTable 2. Association analyses between *APOE* epsilon status and risk of incident ICH in patients with brain arteriovenous malformations of European ancestry

APOE Status	Discovery phase UK Biobank				Replication phase All of Us			
	Univariate logistic regression		Multivariable logistic regression		Univariate logistic regression		Multivariable logistic regression	
	**OR (95% CI)**	**p**	**OR (95% CI)**	**p**	**OR (95% CI)**	**p**	**OR (95% CI)**	**p**
***APOE* Epsilon 4**	2,41 (1,29-4,52)	<0.001	2,42 (1,25-4,73)	<0.001	4.27 (1.39-13.54)	0.01	5.44 (1.55-2.15)	0.01

Abbreviations: UK = United Kingdom; OR = odds ratio; CI = confidence interval

© 2024 Renedo D et al. *JAMA Network Open*.

eTable 3. Association analyses between *APOE* epsilon status and risk of incident intracerebral hemorrhage in patients with brain AVMs in all ancestries

***APOE* Status**	**Discovery phase UK Biobank**				**Replication phase All of Us**			
	**Univariate logistic regression**		**Multivariable logistic regression**		**Univariate logistic regression**		**Multivariable logistic regression**	
	**OR (95% CI)**	**p**	**OR (95% CI)**	**p**	**OR (95% CI)**	**p**	**OR (95% CI)**	**p**
***APOE* Epsilon 4**	1.74 (1.01-2.99)	0.04	1.76 (1.00-3.10)	0.04	1.80 (0.82-3.79)	>0.05	2.-07 (7.60-4.28)	>0.05

Abbreviations: UK = United Kingdom; OR = odds ratio; CI = confidence interval

*All ancestries: included all ancestries in each study (Categories for UK Biobank includes: Caucasian (European Ancestry) and in All of Us included: African/African American, Amercian/Admixed/Latino, East Asian, European, Middle Eastern, South Asia, and Others.)

© 2024 Renedo D et al. *JAMA Network Open*.

eTable 4. Association analyses between *APOE* epsilon status and risk of prevalent ICH in patients with brain arteriovenous malformations of European ancestry

APOE **Status**	**Discovery phase UK Biobank**				**Replication phase All of Us**			
	**Univariate logistic regression**		**Multivariable logistic regression**		**Univariate logistic regression**		**Multivariable logistic regression**	
	**OR (95% CI)**	**p**	**OR (95% CI)**	**p**	**OR (95% CI)**	**p**	**OR (95% CI)**	**p**
APOE **Epsilon 4**	2,40 (1,27-4,53)	<0.001	2,31 (1,20-4,46)	<0.001	3,75 (1.25-11.46)	0.01	4.90 (1.38-19.79)	0.01

Abbreviations: UK = United Kingdom; OR = odds ratio; CI = confidence interval

© 2024 Renedo D et al. *JAMA Network Open*.

eTable 5. Association analyses between *APOE* epsilon status and risk of prevalent ICH in patients with brain arteriovenous malformations of all ancestries

APOE Status	Discovery phase UK Biobank				Replication phase All of Us			
	Univariate logistic regression		Multivariable logistic regression		Univariate logistic regression		Multivariable logistic regression	
	OR (95% CI)	p	OR (95% CI)	p	OR (95% CI)	p	OR (95% CI)	p
***APOE* Epsilon 4**	1,73 (1.02-2.99)	0.04	1,77 (1.01-3.10)	0.04	1.80 (0.82-3.79)	>0.05	2.07 (0.89-4.67)	>0.05

Abbreviations: UK = United Kingdom; OR = odds ratio; CI = confidence interval

*All ancestries: included all ancestries in each study (Categories for UK Biobank includes: Caucasian (European Ancestry) and in All of Us included: African/African American, Amercian/Admixed/Latino, East Asian, European, Middle Eastern, South Asia, and Others.)

© 2024 Renedo D et al. *JAMA Network Open*.

eFigure. Study flow diagram

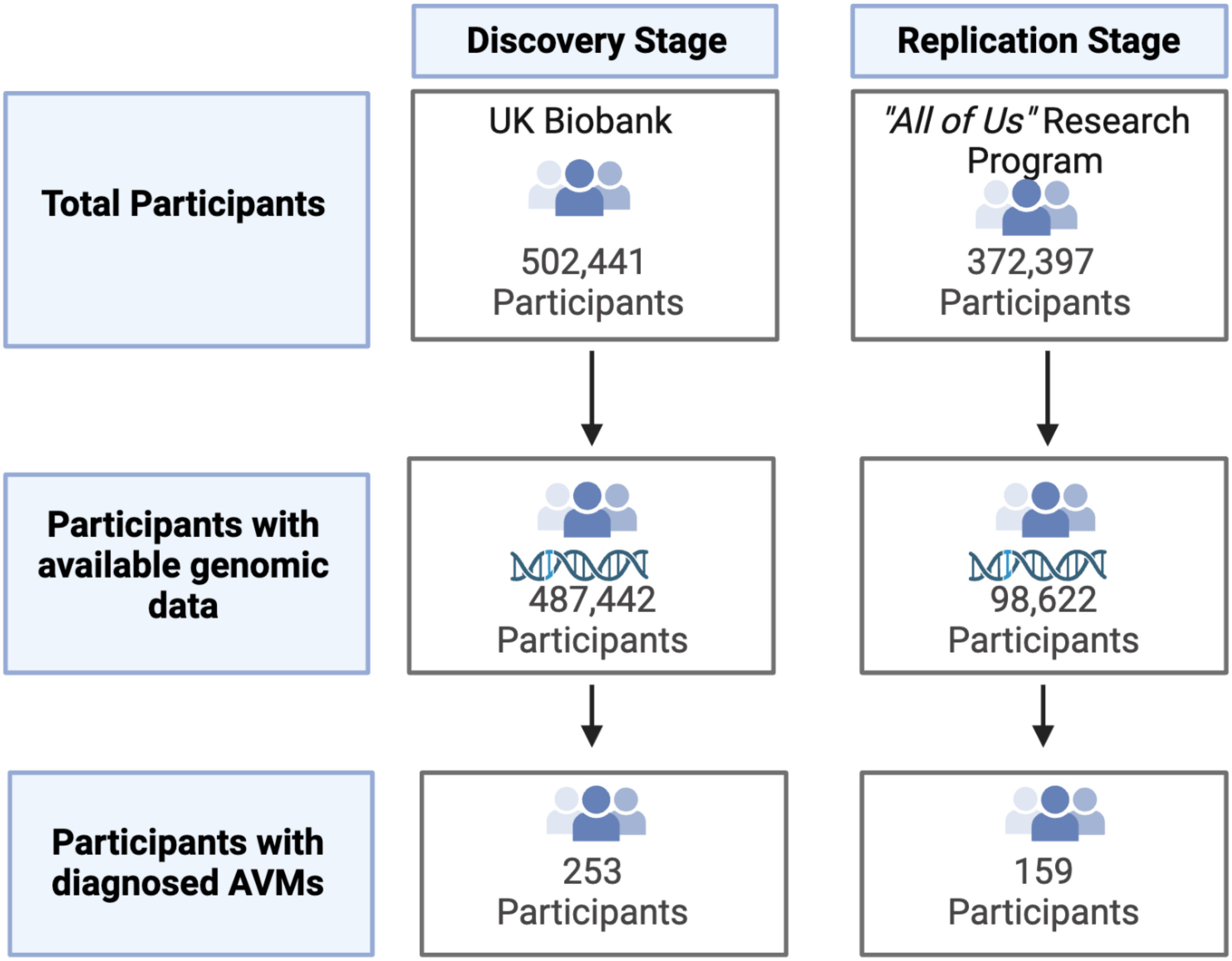

© 2024 Renedo D et al. *JAMA Network Open*.
