# Supplementary material for: APOE ε4 and Intracerebral Hemorrhage in Patients With Brain Arteriovenous Malformation
Source: JAMA Netw Open. 2024 Feb 16;7(2):e2355368. doi: 10.1001/jamanetworkopen.2023.55368 (PMC10873768; doi:10.1001/jamanetworkopen.2023.55368)
Supplement: Supplement 2. — Data Sharing Statement [file jamanetwopen-e2355368-s002.pdf]

Data Sharing Statement

Renedo. APOE ε4 and Intracerebral Hemorrhage in Patients With Brain Arteriovenous Malformation. *JAMA Netw Open*. Published February 15, 2024.
doi:10.1001/jamanetworkopen.2023.55368

Data
Data available: Yes
Data types: Other (please specify)
Additional Information: Data is available through the All of Us Research Program (https://allofus.nih.gov/) and the UK Biobank (https://www.ukbiobank.ac.uk/)
How to access data: https://allofus.nih.gov/ https://www.ukbiobank.ac.uk/
When available: With publication

Supporting Documents
Document types: None

Additional Information
Who can access the data: Researchers whose proposed use of the data has been approved by All of Us or UK Biobank
Types of analyses: any purpose
Mechanisms of data availability: signed data access agreement without investigator support,
